# Spatial Heterogeneity in Response of Male Greater Sage-Grouse Lek Attendance to Energy Development

**DOI:** 10.1371/journal.pone.0097132

**Published:** 2014-06-11

**Authors:** Andrew J. Gregory, Jeffrey L. Beck

**Affiliations:** 1 School of Earth, Environment and Society, Bowling Green State University, Bowling Green, Ohio, United States of America; 2 Department of Ecosystem Science and Management, University of Wyoming, Laramie, Wyoming, United States of America; Roehampton university, United Kingdom

## Abstract

Landscape modification due to rapidly expanding energy development, in particular oil and gas, in the westernUSA, have prompted concerns over how such developments may impact wildlife. One species of conservation concern across much of the Intermountain West is the greater sage-grouse (*Centrocercusurophasianus*). Sage-grouse have been petitioned for listing under provisions of the Endangered Species Act 7 times and the state of Wyoming alone represents 64% of the extant sage-grouse population in the eastern portion of their range. Consequently, the relationship between sage-grouse populations and oil and gas development in Wyoming is an important component to managing the long-term viability of this species. We used 814 leks from the Wyoming Game and Fish Department's lek survey database and well pad data from the Wyoming Oil and Gas Conservation Commission to evaluate changes in sage-grouse lek counts as a function of oil and gas development since 1991.From 1991–2011 we found that oil and gas well-pad density increased 3.6-fold across the state and was associated with a 24% decline in the number of male sage-grouse. Using a spatial and temporally structured analysis via Geographically Weighted Regression, we found a 1-to-4 year time lag between development density and lek decline. Sage-grouse also responded to development densities at multiple spatial neighborhoods surrounding leks, including broad scales of 10 km. However, sage-grouse lek counts do not always decline as a result of oil and gas development. We found similar development densities resulting in different sage-grouse lek count responses, suggesting that development density alone is insufficient to predict the impacts that oil and gas development have on sage-grouse. Finally, our analysis suggests a maximum development density of 1 well-pad within 2 km of leks to avoid measurable impacts within 1 year, and <6 well-pads within 10 km of leks to avoid delayed impacts.

## Introduction

Anthropogenic impacts to nature are nearly omnipresent across Earth [1]. Today, human-caused fragmentation and land use are the main threats to biodiversity conservation [2], [3]. Recent expansion of energy development throughout the Intermountain West has prompted concern about how such developments might impactprairie-grouse including greater sage-grouse (*Centrocercusurophasianus;* hereafter sage-grouse) populations [4]. The current distribution of sage-grouse encompasses 11 western states and 2 Canadian provinces and is tied to the presence of sagebrush (*Artemisia* spp.; [5]). Declines in the distribution and abundance of sage-grousehave been a concern since at least the early 1900s [6].However, this decline has increased across the range of sage-grouse over the last 50-years [7], prompting 7 separate petitions to listsage-grouse under provisions of the Endangered Species Act (ESA) of 1973. In the most recent attempt in 2010, the U. S. Fish and Wildlife Service (USFWS) found that listing greater sage-grousewas warranted, but precluded by higher priority listings for other species [8]. Concerns persist as current data suggest that over the last 50 years, human demand for energy has increased by >50% and a similar increase is anticipated by 2030 [9].

Several recent studies investigating the possible influence of oil and gas development on sage-grouse lek attendance have found that sage-grouse are negatively impacted by oil and gas development [4], [10]–[15]. However, these studies evaluated the response of sage-grouse at local scales (e.g. basin or development site) or quantified the direction and magnitude of the impacts over broad geographic expanses that didnot account for possible spatial variation in response. It is possible that in some areas and under certain conditions, sage-grouse might be coping with oil and gas development, and so it is important to identify these areas and conditions so that we can better understand what characteristics of these locations are driving these trends.

A listing decision for the greater sage-grouse under the ESA would have severe economic impacts for thestates where sage-grouse are found. For example, Wyoming contains 64% of the sage-grouse in the eastern portion of the species range [16], and is an energy-rich state, with large reserves of coal, natural gas, oil, uranium, and high potential for wind energy development [17], [18]. Sage-grouse distribution in Wyoming overlaps with high priority energy development sites, including those for oil and gas [19]. In recognition of this, the state of Wyoming instituted a regulatory approach to conserving sage-grouse through an executive order issued by the governor. This Sage-grouse Executive Order (SGEO) established sage-grouse core areas, limiting development in areas of high priority for sage-grouse [20]–[22].

Previous studies on the potential impacts of oil and gas development on sage-grouse lek attendance have typically modeled raw lek count data or a binomial characterization of lek occupancy as either occupied or unoccupied as a response variable [10], [12], [13], [15].When dealing with data collected at spatially discrete locations or interpolated across spatially connected areas one expects there to be a high degree of spatial autocorrelation [23]. Moreover, because of some of the unique properties of spatially structured observations, near objects ought to be more related than distant objects [24]. Consequently, the assumption made by ordinary least squaresregression techniques that all observations are independent or nearly independent is violated and therefore the application of such techniques to spatially structured data is questionable [25]. Spatial regression controls for the lack of independence in observations by applying a spatial weights matrix to the response variables [26]. Whereas this approach does control for the effects of spatial autocorrelation, it masks possibly interesting and important local data structure that may exist and may average important local trends with a global average [27]. For example, relying on the average temperature for the United States would not be a helpful way to decide if one needs to wear a jacket on any given day in any given region of the United States. Geographically Weighted Regression (GWR) was designed for just this purpose, to control for global spatial autocorrelation in spatially structured data sets, while still maintaining local variation and patterns that might be important drivers of the system (i.e., spatial heterogeneity; [27].

Our primary objective was to explore the possibility of spatially varying relationships among oil and gas development density and sage-grouse lek attendance. Previous studies, working within important sage-grouse habitats in Wyoming, have suggested that such a relationship might exist. Doherty et al. [14] identified 17 leks in Wyoming that persisted despite a high development density of ≥40 wells within 32.2 km^2^ of these leks, and via visual inspection of these leks suggested that on 11 of them oil and gas development was clustered leaving large open spaces. However, Doherty et al. 's analysis focused on lek persistence over a 4-year period, and over that period these 17 leks declined by ∼55% [14]. Thus, our secondary objective was to investigate the possibility of similar development densities resulting in opposite trends in sage-grouse lek attendance, in an effort to understand if there is a sustainable development density of oil and gas well pads where lek attendance is not impacted. To obtain data to address our objectives we used a regression trend analysis of male sage-grouse lek count data for the state of Wyoming as an index of the statewide sage-grouse population trend. We modeled sage-grouse population trends for the most recent 3, 5, and 10 year time periods since 2011 as a function of oil and gas well-pad density at multiple spatial neighborhoods around leks. We also evaluated the effects of past well-pad densities on the current sage grouse lek count response to oil and gas development in an attempt to assess lag effects.

## Methods

### Terminology used throughout the manuscript

Throughout this article we refer to our response variable, based on Wyoming Game and Fish Department (WGFD) lek count data—as lek attendance. Previous studies using the same database have used the term sage-grouse population trends [13], [19], [28]. We hesitate to use the terms abundance or population trajectory as the lek-count data estimates we used do not account for detection probabilities ≠1.0, which is necessary to truly estimate abundance or a population trajectory [29]. In addition, we used lek count values for only peak male attendance so females were not included in our estimates. Thus, we prefer to use the term male lek attendance.

### Sage-Grouse Lek Data

We used WGFD annual sage-grouse lek survey count data to index sage-grouse population trend (T. Christiansen, WGFD Sage-Grouse Program Coordinator, personal communication, 2010). The WGFD lek database identifies a lek as a traditional courtship display area in or adjacent to sagebrush-dominated habitat attended by ≥2 male sage-grouse for two or more consecutive years (WGFD Sage Grouse definitions 2010). The database contains geographic coordinates and annual count data since 1948 for leks across Wyoming. However, the majority of these leks, 68%, do not have regular survey data (an average of ≥3 out of 5 years) until after 1973. Lek counts were conducted by WGFD,other natural resource agency personnel, or trained volunteersfollowing established WGFD protocol. Only ground counts were used in our analysis. In each given year, surveyed leks were categorized as active, inactive, or unknown. On all active leks numbers of male and female sage-grouse present on the lek at the time the survey was conducted wererecorded. What this means, is that both male and female sage-grouse were counted, but, due to the fact that adult males attend leks consistently throughout spring [30], and female lek attendance is inconsistent, we elected to use the count data for only peak male attendance, which is consistent with previous analyses [11], [12].Leks were classified as occupied based on presence of strutting males at least once during the most recent 10 years. Leks that were surveyed only once were also included in our analysis because for analyses with >50 sage-grouse leks, repeated counts are not necessary to model population trend estimates [28]. Based on the annual lek survey data, leks were assigned a management status based on whether the lek site was observed to have had birds displaying on it sometime during the most recent 10-years as either occupied or unoccupied.

**Figure 1:**
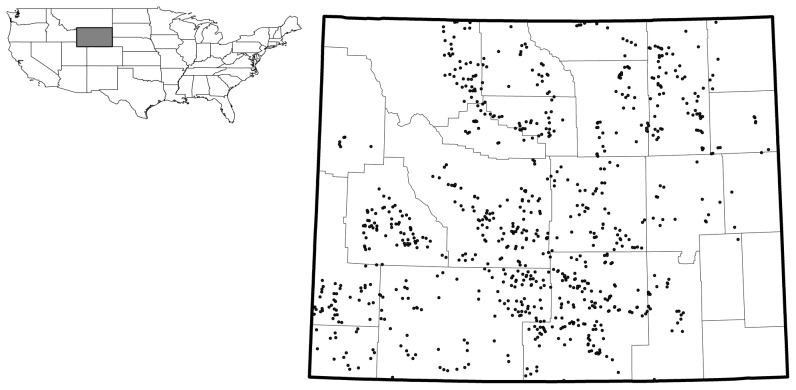
Point locations of 814 sage-grouse lek sites used in the GWR analysis, from 2002–2011, Wyoming USA. Inset boundaries outline Wyoming's 23 county boundaries.

We used data from the WGFD sage-grouse lek survey database for 2002 to 2011. To be included in our analysis,sage-grouse leks had to have been surveyed in all years from 2009–2011, in at least 4 years from 2007–2011including all years from 2009–2011, and in at least six years from 2002–2011 including surveys in 2002 and in all years from 2009–2011. This restriction was necessary to allow us to characterize a regression of lek count data across each time period (most recent 3, 5, and 10 years, respectively). These restrictions left us with a database of 814 leks counted a total of 4,070 times in ten years (average of 5/lek) across Wyoming for our analysis (Figure 1).

### Well Pad Data

Oil and gas well-pad density data were summarized from the Wyoming Oil and Gas Conservation Commissions database of Wyoming Statewide Oil and Gas Drilling Activity to 2010 [31]. The database contains geographic coordinates and attribute data for all (114,246) oil and gas wells in the state of Wyoming from 1914–2010. All well-padswere assigned to 1 of 11 classifications and 1 of 29 statuses delineating the type of well pad and the amount of activity present at each well pad site [31]. Our well-pad density estimates are for active well-pads each year.

**Figure 2:**
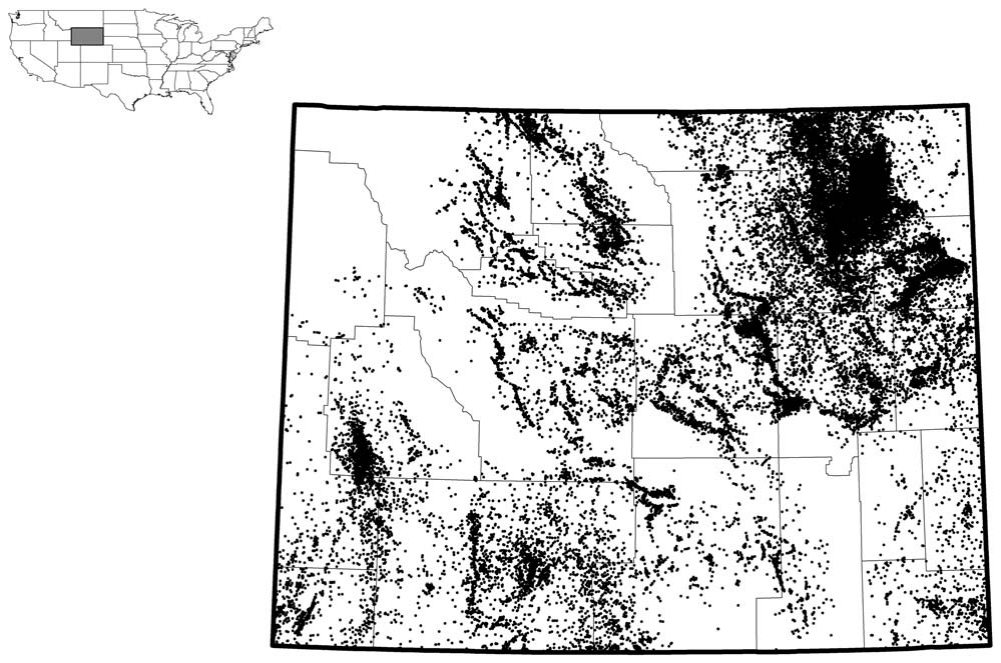
Point locations of 39,885 active oil and gas well pads from 1991–2011 in Wyoming, USA used to create well pad density maps.

For the purposes of our study, well pads were classified according to the presence of physical structures on the landscape and of a status indicating that they were actively producing over the time period for which sage-grouse data were collected [31]. For example, some well pads had a classification AP (Permit to Drill), but no indication that drilling had ever occurred; so these well-pads were not included in our analysis. In other instances a well pad might have a status of DH (dry hole) or DR (dormant) accompanied by a date for when activity at the site ceased. These well-pads were also not included in our estimate of well-pad densities. Eliminating these inactive or undeveloped well pads provided us with a geodatabase that included location data for 39,885 active well-pads for the state of Wyoming (Figure 2).

**Figure 3:**
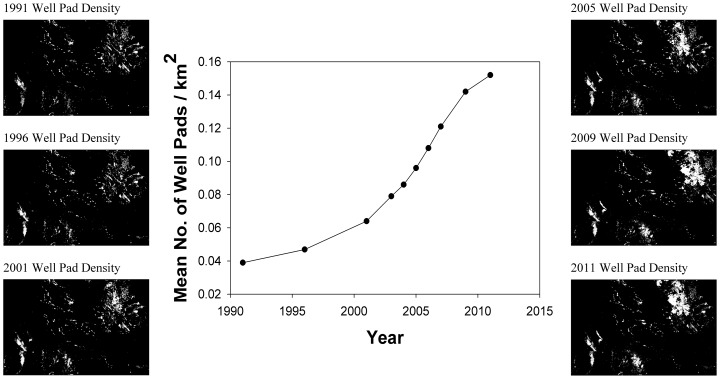
Well pad density maps created from the WOGCC well pad data from 1991–2011, Wyoming, USA. Maps depict relative well pad densities (number of well pads/km^2^) across Wyoming from 1991–2011. White areas indicate areas of higher well pad density. Values range from 0 well pads/km^2^ to 50.6 well pads/km^2^, which was the highest observed density in 2011. Well pad densities plotted along the line plot, are the average well pad densities across Wyoming. Thus, the 3.6-fold increase in observed well pad density from 1991–2011 suggests not only an intensification of development within developed areas, but also an increase in area that was under development for oil and gas production.

We used a roving window analysis to calculate the point density of well-pads within 0.5, 1, 2, 5, and 10 km areas surrounding leks in Arc Info 10.1 (Environmental Systems Research Inc., Redlands, California). Well pad densities were calculated for all well-pads present on the landscape for all years prior to 2009, 2007, 2006, 2004, 1996, and 1991 to test for possible existence of 1-, 3-, 5-, and 10-year time lags to population effects, respectively (e.g., Figure 3).

### Statistical Analysis

Sage-grouse lek count data shows periodicity [28]. While over 10- and 20-year periods the general trend (increasing, decreasing, or stable) is accurate, over 3- to 5-year periods within the 10- and 20-year periods, count data will suggest that populations at leks alternate from exhibiting an increasing trend to decreasing trend [28]. To control for this periodicity we used a logit function calculated from the 10-year LOWESS regression to back calculate lek attendance values for each year of the 10-year period [32]. The LOWESS function is a smoothing function used to detrend data and to dampen the effect of outliers on the data set [33]. We used a heuristic algorithm to choose a smoothing factor for the LOWESS function based on the AIC_C_ Information Criterion [34]. We then applied a linear regression to the 10-year LOWESS predicted lek counts for the most recent 3, 5, and 10 year periods from 2011.We refer to these responses as the 3×10, 5×10, and 10×10 sage-grouse responses. We used parameter estimates from the 3-,5-and 10-year regressions possible sage-grouse responses trends to oil and gas development. Finally, we also used the raw LOWESS adjusted count data from 2011 and a binomial response variable of increasing (1) or decreasing (0) as response variables for each lek so that we could compare our analytical results with previously published studies.

We used GWR to assess the impact that oil and gas well-pad density had on male sage-grouse lek attendance and to test for possible spatial heterogeneity in the response of sage-grouse to oil and gas development [27].An advantage of the GWR approach is that it evaluates localized patterns in the response variable to localized patterns of predictor covariates. Thus, localized patterns in response are accounted for in a single analytical procedure [27]. This was an important component of this analysis as we expected different patterns in sage-grouse response to oil and gas development across our Wyoming study system, specifically when comparing the northeast to southwest portions of the state (T. Christiansen, WGFD Sage-Grouse Program Coordinator, personal communication, 2013).

We calculated separate GWR analyses for each response variable; thus, we had 5independent sets of GWR model results (3×10, 5×10, 10×10, 2011 Count, and Binomial). For predictor variables we used well pad densities at 0.5, 1, 2, 5, and 10 km surrounding leks, and tested for time lags of 1, 3, 5, and 10 years. These values were chosen based on observations that previous studies had found impacts at such temporal and spatial scales [11]–[14]. We used goodness-of-fit tests and model significance (Pseudo-R^2^) values to rank suitability of response variables. A note about pseudo-R^2^ values: pseudo-R^2^ values are not the same as R^2^, and cannot be directly interpreted as proportion of explained variance, their interpretation is actually more similar to an AIC value except that the larger the value the more support for that particular model; assuming that the data set from which the models are derived have similar magnitudes and variance [35].A second difference is that pseudo-R^2^ values are not bounded 0-1, they can be negative and they can be >>1.0 [35]. This later issue is a particularly salient point to consider when dealing with pseudo-R^2^ values generated via a Monte Carlo simulation procedure as was done for our analysis within GWR [27]. Thus, comparisons of pseudo-R^2^ values to determine strength of model support are only appropriate for models using as a response variable the regression trends across LOWESS transformed point estimate (Table 1).

**Table 1:** Results of GWR analyses with each response factor for the most parsimonious period over which the impacts of oil and gas development were observed onsage-grouse lek attendance,2002–2011, Wyoming, USA.

Model	Leks	No. Parameters	Pseudo-R^2^
5×10	814	47.8	0.32
10×10	814	30.8	0.07
3×10	814	65.9	0.17
*Binary active/inactive	1,298	31.3	0.13
*2011 lek count	2,012	1,276.0	451.20

*Analyses were conductedon data sets with different number of leks, data

magnitudes, and unequal variance structures to LOWESS adjusted models; thus

Psuedo-R^2^ value comparison to LOWESS/regression models is not appropriate.

Models are presented for comparison purposes only.

With the most supported modelwe used *post hoc*Monte Carlo procedures for goodness-of-fit tests to test for significant impacts of predictor variables that were driving model performance [36]. We generated parameter estimates and *Z*-scores for each predictor covariate at each lek point. We then mapped predicted trends in sage-grouse lek count values back onto the oil and gas development density maps of Wyoming for only those leks that had a significant response to the specified oil and gas development density. We then used inverse distance weighting spatial interpolation as implemented in Arc Info 10 (Environmental Systems Research Institute, Redlands, CA; [37]) to create a map of sage-grouse heterogeneic response to oil and gas development density by year that were identified as important in the most supported model.

Finally, we used the functional response curve of sage-grouse lek attendance to oil and gas development densities to forecast predictions about how sage-grousewill likely respond in the future to current development densities. This was done to aid in setting regional management recommendations for sage-grouse in light of ongoing oil and gas extraction within sage-grouse core areas in Wyoming. All statistical analyses were conducted in either Program R [38] or Program GWR [36].

## Results

**Figure 4:**
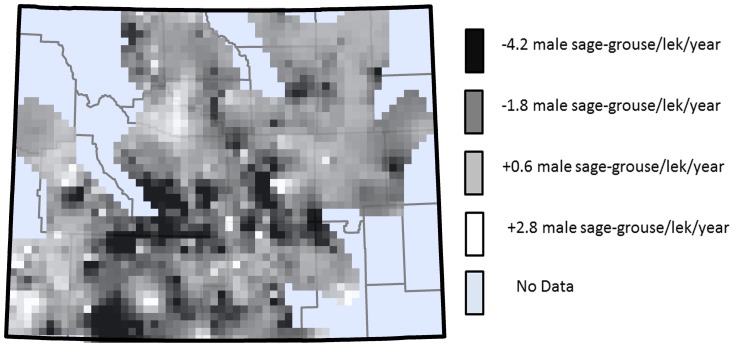
Spatial pattern in male sage-grouse lek declines from 2007–2011 across Wyoming, USA.

From 1991–2011 we observed a 3.6-fold increase in the median well pad density across Wyoming (Figure 3). Over this sameperiod,at the 814 leks in our analysis,we observed a 23.8% decline (from 18,631 to 14,185 male sage-grouse) in male sage-grouse lek attendance. However, spatial analysis suggested that the rate of loss was not uniform across the state, and ranged from –4.2 males/lek/year average loss to +2.8 males/lek/year average gainacross the 20 years of our analysis at our 814 leks (Figure 4). It is important to note that while some leks were observed to increase, these were exceptions and not the norm.

We specifically used GWR to identify the appropriate response period and region over which a particular oil and gas development density had the strongest impact on male sage-grouse lek attendance. Analysis with GWR suggested that the5-year response period (adjusted for periodicity with LOWESS) form 2007–2011 showed the strongest response to oil and gas development (*F* = 4.16, *P* = 0.042; Table 1). The 5-year response period GWR analysis suggested that a model including spatial heterogeneity had 2.2-fold better performance than a spatial regression model that did not take spatial heterogeneity of response factors into consideration (spatial regression AIC_C_  = 9,327; GWR AIC_C_  = 4,238). *Post hoc* Monte Carlo significance tests indicated that there were 4 predictor covariates with a significant impact on the spatial heterogeneity of the GWR model performance (Table 2). Specifically, the Monte Carlo analysis suggested that heterogeneity in male sage-grouse lek attendance was most strongly associated with development density within 1, 2, and 10 km of the lek, 1 and 4 years (2006 and 2003, respectively) prior to the start of our 5-year response period.

**Table 2:** GWR *post hoc* Monte Carlo analysis of covariate influence on GWR model performance, 1996–2007, Wyoming, USA.

Time Lag x Well Pad Density	Parameter Estimate	Monte Carlo *P*
Intercept	−2.35 to −1.13	0.00
10 year; 10 km	−18.44 to −11.12	0.50
10 year; 5 km	−4.83 to −2.84	0.88
10 year; 1 km	−8.94 to −4.95	0.50
10 year, 500 m	−2.93 to −1.58	0.56
*4 year; 10 km	−19.40 to −12.46	0.02
4 year; 5 km	−22.32 to −11.05	0.30
*4 year; 1 km	−1.32 to −2.79	0.03
4 year; 500 m	−1.50 to −0.45	0.56
1 year; 10 km	−13.24 to −6.05	0.21
1 year; 5 km	−28.11 to −17.52	0.42
*1 year; 1 km	−2.79 to −1.32	0.00
*1 year; 2 km	−5.00 to −3.02	0.04

* Indicates parameters that had a significant influence on GWR model performance and thus where further analysis of the regional variation in covariate performance was informative.

**Figure 5:**
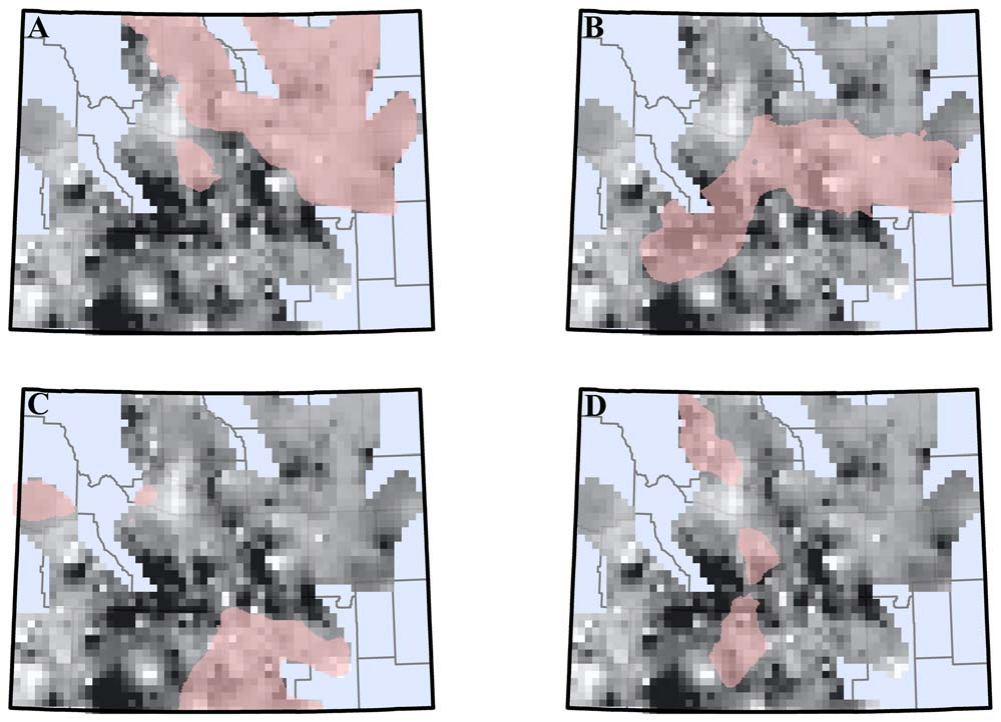
Spatial variation in response of male sage-grouse lek counts from 2007–2011 to increases in oil and gas well pad development density from 1996–2011, across Wyoming, USA. Lag effect in lek attendance was 1 year for the 2006 temporal scale and 4 years for the 2003 temporal scale. A  =  spatial region where 2007–2011 male sage-grouse lek attendance was significantly associated with well pad development density within 1 km of leks in 2006. B  =  spatial region where 2007–2011 male sage-grouse lek attendance was significantly associated with well pad development density within 2 km of leks in 2006. C  =  spatial region where 2007–2011 male sage-grouse lek attendance was significantly associated with well pad development density within 1 km of leks in 2003. D  =  spatial region where 2007–2011 male sage-grouse lek attendance was significantly associated with well pad development density within 10 km of leks in 2003. Average well pad density for A  =  0.14 well pads/km^2^. Average rate of lek attendance change for A  =  −1.89 males/lek/year. Average well pad density for B  =  0.14 well pads/km^2^. Average rate of lek attendance change for B  =  1.63 males/lek/year. Average well pad density for C  =  0.1 well pads/km^2^. Average rate of lek attendance change for C  =  −0.82 males/lek/year. Average well pad density for D  =  0.5 well pads/km^2^. Average rate of lek attendance change for D  =  −2.29 males/lek/year.

Based on the results of the Monte Carlo analysis, we examined individual lek response to oil and gas development density at 1, 2, and 10 km. For each individual lek, GWR computes an independent *t*-test of significance. We used an inverse distance weighted function to characterize the spatial distribution over which development density at each significant parameter (1, 2, and 10 km and 1 and 4 year time lags) had a significant localized response (Figure 5). We then used the 5-Year LOWESS functional response for each lek and back calculated to determine the rate of change in terms of number of males per lek per year that were associated with development density in each region (Figure 5). Based on the observed rates of change in male sage-grouse lek attendance observed for each development density and time lags (Figure 5), we extrapolated what the expected response would be given current oil and gas well pad development density.

To extrapolate the effect of well pad density on male sage grouse lek attendance we created plots of observed lek attendance versus observed well pad density for each focal region surrounding leks and then calculated a trend line for each data plot. For development density within 1 km of leks with a 1-year time lag, the trend line was:*change in male lek attendance*  =  −1.45×well pad density −3.3. For development density within 2 km of leks with a 1-year time lag, the trend line was:*change in male lek attendance*  =  −5.98 ×well pad density −0.52. For development density within 1 km of leks with a 4-year time lag, the trend line was:*change in male lek attendance*  =  −7.7× well pad density +3.02. Finally, for development density within 10 km of leks with a 4-year time lag, the trend line was:*change in male lek attendance*  =  −2.1× well pad density −0.02. In2011, the average well pad density within 1 km of leks was 0.08 well pads/km^2^, within 2 km of leks the average well pad density was 0.13 well pads/km^2^, and within 10 km of leks the average well pad density was 0.17 well pads/km^2^.

## Discussion

From 1991–2011 we observed a 3.6-fold increase in the median well pad density across Wyoming, which was spatially associated with a 23.8% decline in the number of male sage-grouse counted at leks. During about this same time period (1997–2007) a range-wide sage-grouse lek count analysis of 3,679 leks in seven sectors indicated that 44% of leks declined, whereas 56% of leks increased [39]. Distance to nearest oil and gas development site was included as a possible explanatory covariate in this analysis and the two sectors in Wyoming had a distance to nearest oil and gas development an order of magnitude closer than in other sectors and also the largest declines in sage grouse lek count [39]. Our study in conjunction with the Johnson et al. [39] study suggests a dramatic decline in sage-grouse populations in Wyoming that is at least partially due to intense oil and gas development in that state.

Our findings indicated that male lek attendance across Wyoming varied spatially and temporally with the density of oil and gas well pads. We also identified similar development densities wherein male sage-grouse lek attendance increased or decreased, that is there were a few leks where male lek counts remained stable or actually increased slightly in spite of high density oil and gas development. As with other studies, our analysis indicated that a 4-to-5 year lag occurs between the time that oil and gas development reaches a particular density to when population-level sage-grouse responses are observed [12]–[14]. This lag period makes sense in terms of sage-grouse philopatry to lek sites and sage grouse biology. Although some males recruit to a lek at year one, in general it takes 2-3 years for male sage grouse to recruit to a lek and once they recruit they have relatively high adult survival as well as high philopatry to the lek site [7], [12], [13]. If oil and gas development leads to greatly reduced recruitment to a lek, but not complete reproductive failure, then philopatry to that lek and high annual survival of adult males ensures a relatively stable lek for several years into the future, and hence a lag to an observable impact on lek count. We also identified spatial regions where particular oil and gas development densities were having a significant impact on sage-grouse populations.

From 1991 to 2011 we observed a nearly 4-fold increase in oil and gas well-pad density across the state of Wyoming. In addition to the observed impacts on male sage-grouse lek attendance reported in this paper, such an increase in oil and gas development may also have incurred population-level impacts on other rangeland species. For example,oil and gas development in western Wyoming reduced mule deer (*Odocoileushemionus*) use of wintering habitat [40], [41], and led to declines in abundance of sagebrush obligate songbirds [42]. Understanding and mitigating the effects of oil and gas development density for sage-grousemay also provide benefits for these other species, as sage-grouse are typically described as a landscape [7], [39]or umbrella [43] species.

In our analysis, we observed that oil and gas development had both a different spatial and temporal scale of impact on male sage-grouse lek attendance. By evaluating the observed development density for those leks in our analysis with observed declines, we can better understand the influence that oil and gas development has on lek persistence. For example, our analysis suggests that leks in the northeastern portion of the state had an immediate (within 1 year) response to oil and gas development density within 1–2 km of the lek site (Figures 5A,B). One reason for this is likely due to lek size, in terms of number of males attending the leks in this region. Leks in this region were small, typically 4–8 birds (WGFD lek database data). Using the link function we were able to back calculate from our observed rates of decline in this region of 25–80% to an average decline of 1–4 males/lek/year (Figure 4). Conversely, in the southwestern portion of Wyoming, leks were typically larger (12^+^ males/lek; WGFD lek database data). At small leks (4–8 birds), a 20–25% decline is likely a much more severe loss than is a similar rate of decline at large leks (12–20 birds). Moreover, at a small lek, a loss of a single bird will necessarily also account for a larger percent decline in lek attendance. Thus, high rates of decline noted in northeastern Wyoming, while representing only a small portion of the total state-wide sage-grouse population, may still be of significantly greater concern than observed rates of decline at other regions of this analysis because they could lead to more rapid extirpation of the species from that region. Specifically, we observed immediate effects of oil and gas development density on male sage-grouse lek attendance based on significant declines in attendance associated with development density occurring within 1 km and 2 km of leks in 2006, 1 year prior to our analysis period. Over this time frame, male sage-grouse lek attendance declined in areas where well-pad densities exceeded 0.19 well-pads/km^2^ within 1 km of leks, and ≥2.13 well-pads/km^2^ within 2 km of leks, particularly in the northeastern portion of Wyoming (Figures 5A,B). Consequently, our data suggest that to avoid immediate declines in male sage-grouse lek attendance, no active oil and gas well-pads should be placed within 2 km of leks. This is particularly true in the northeastern portion of Wyoming where lek sizes are typically small, and lek attendance responds rapidly to oil and gas development density. This has important management implications, as previous work has shown that reduction in lek attendance due to energy development in this region may increase sage-grouse susceptibility to other stressors such as West Nile Virus [44].

In addition, we also observed a temporal lag-affect in male sage-grouse lek attendance associated with oil and gas development. Specifically, we observed that the 5-year trend in male sage-grouse lek attendance was responding to oil and gas development that had occurred 4 years prior at two different spatial scales of 1 km and 10 km. What this means is that when we evaluated the trend in male sage-grouse lek attendance from 2007–2011 it responded strongly to the well-pad density that was present on the landscape in 2004. It also suggested that the spatial area over which sage-grouse were sensitive to oil and gas development was both local, within 1 km of leks, and broad, within 10 km of leks. Thus, leks with significant declines in male sage-grouse attendance had oil and gas development densities ≥0.06 well-pads/km^2^ within 1 km of leks and ≥0.7 well-pads/km^2^ within 10 km of leks.Leks with oil and gas development densities at or above these levels are expected to show declines in male lek attendance 4–9 years after development occurs. Previous studies have also noticed similar lag periods to effect [12], [13], and presumably this lag effect is related to the time needed for juvenile sage-grouse to mature to reproductive age and recruit to a lek [13]. Because well-pad development density ≥0.7 well-pads/km^2^ (7 well-pads within 10 km of the active lek site) resulted in significant declines in male lek attendance 9years after development, our data suggest that to avoid long-term declines, possibly associated with failed recruitment, no more than 6 well-pads should be placed within 10 km of active leks.

We also identified 51 lek locations where identical oil and gas well-pad development density resulted in opposing male lek attendance responses, such that some leks declined, whereas others leksremained stable or increased. The next step in identifying why lek attendance at these leks responded differently to equal intensities of energy development is to evaluate what differences exist in these areas such as development configuration, climate, and land management and evaluate what effect these characteristics have on male sage-grouse lek attendance [14], [15].One possible explanation is that differences in well-pad development configuration may result in different sage-grouse lek response to similar overall development density. For example, over a 1,000 ha area, dispersed configurations may lead to high-localized development density, such that every lek is close to at least one and possibly several well pads. Over that same area, clustered configurations would likely lead to some leks being in an area of very high density (6–10 well pads), but most leks being far from any well-pads [14]. This is an intriguing possibility and the degree to which this is true and the consequences for sage-grouse needs to be examined in greater detail.

**Figure 6:**
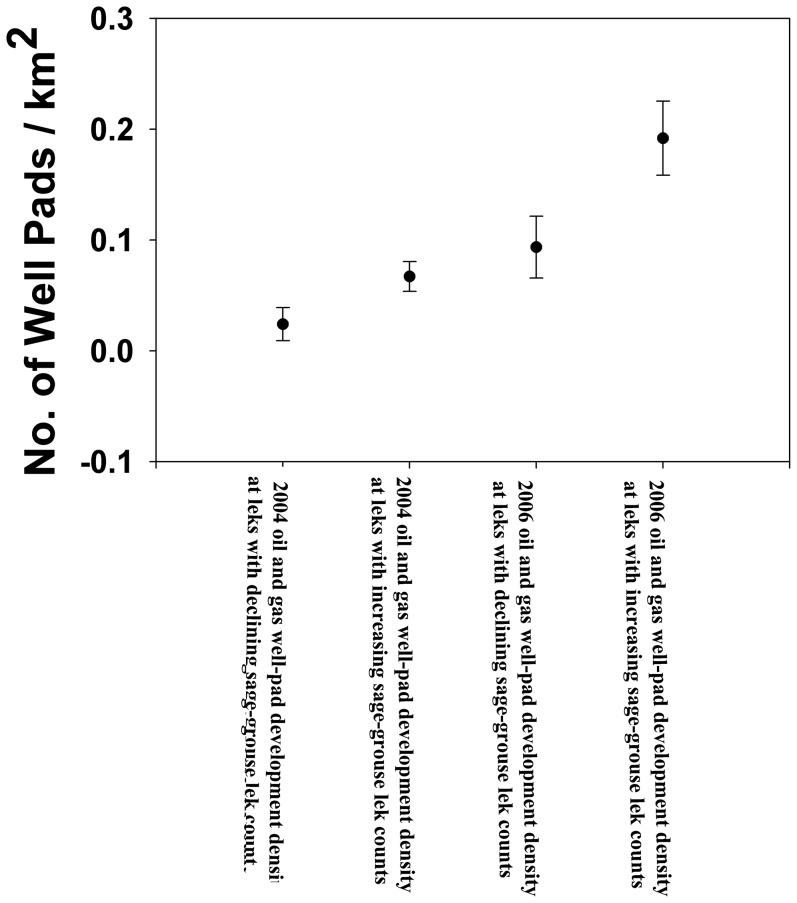
Variation (mean ± SE) in male sage-grouse lek response from 2007–2011 to identical development density, in 2004 (4-year time lag) and 2006 (1-year time lag), Wyoming, USA. In 2004 the average sage-grouse lek increase was +3.2 males/lek/year (n = 36 leks) and the average sage-grouse lek decline was −5.7 males/lek/year (n = 153 leks). In 2006 the average sage-grouse lek increase was +3.3 males/lek/year (n = 42 leks) and the average sage-grouse lek decline was −4.9 males/lek/year (n = 125 leks).

In addition, based on current development density and the lag to the effects observed for the 814 leks in our analysis, we predict an average of 5.09 male sage-grouse/lek/year decrease over the next 4 years (2012–2015) across Wyoming. Local weather patterns, management actions, and year-to-year variation in sagebrush habitat productivity will undoubtedly affect this predicted rate of loss up or down (*see* [45]). This rate of loss is still sobering, as it suggests that based only on the current development density as of 2011, up to 24% of the 2011 male sage-grouse population may be lost in a short time. However, even though the average response in all regions was negative, not all leks in a given region declined. Moreover, the same development density did not always result in the same functional response in male lek attendance. In 51 instances, identical development densities resulted in very different lek responses (Figure 6). However, it is important to note that development density at these leks may also have had a negative impact as it is possible that some of these leks which maintained stable lek attendance in light of current development density may have in the absence of development increased. While it is impossible with our analysis to directly assess this, it is an intriguing possibility.

Wyoming has undertaken efforts since 2007 under their Sage-Grouse Executive Order (SGEO) to develop regulatory mechanisms within “Core Areas” to provide protection and conservation forsage-grousewithin the State [20]. Then Governor Freudenthal formed a sage-grouse implementation team that recognized 31 sage-grouse“Core Areas” that encompassed approximately 24% of the surface area of the State and provided protections for 82% of the breeding population of sage-grouse (Wyoming Game and Fish Department, Cheyenne, unpublished data). Regulatory mechanisms that direct development within analysis areas established in Core Areas include (1) the number of surface disturbances is notto exceed a density of 1 per 2.6 km^2^averaged across the disturbance analysis area, (2) total accumulated surface area affected (both existing and proposed) within an analysis area is not to exceed 5%, and (3) permanent surface disturbances may not occur within 1 kmof any active or occupied sage-grouse lek [22].Our finding that sage-grouse lek attendance was negatively impacted by as few as 1 well pad within 2 km of sage-grouse suggests that management for sage-grouse based on regulatory mechanisms provided in the Wyoming SGEOmay represent the absolute maximum sustainable development density.Moreover, secondary findings from our study indicate different spatial configurations of well pads that do not influence lek persistence need to be examined more fully to identify disturbance levels that may be more harmonious with leks inside and outside Core Areas.
